# Active Ground Optical Remote Sensing for Improved Monitoring of Seedling Stress in Nurseries

**DOI:** 10.3390/s100402843

**Published:** 2010-03-29

**Authors:** Jan U. H. Eitel, Robert F. Keefe, Dan S. Long, Anthony S. Davis, Lee A. Vierling

**Affiliations:** 1 Geospatial Laboratory for Environmental Dynamics, University of Idaho, PO Box 441133, Moscow, ID 83844-1133, USA; E-Mail: leev@uidaho.edu; 2 Center for Forest Nursery and Seedling Research, University of Idaho, PO Box 441133, Moscow, ID 83844-1133, USA; E-Mails: rkeefe@vandals.uidaho.edu (R.F.K.); asdavis@uidaho.edu (A.S.D.); 3 Columbia Plateau Conservation Research Center, PO Box 370, Pendleton, OR 97801, USA; E-Mail: dan.long@ars.usda.gov

**Keywords:** chlorophyll a+b concentration (Chl_ab_), Scots pine (*Pinus sylvestris*), red-edge, NDRE, NDVI, CCCI, crop sensors

## Abstract

Active ground optical remote sensing (AGORS) devices mounted on overhead irrigation booms could help to improve seedling quality by autonomously monitoring seedling stress. In contrast to traditionally used passive optical sensors, AGORS devices operate independently of ambient light conditions and do not require spectral reference readings. Besides measuring red (590–670 nm) and near-infrared (>760 nm) reflectance AGORS devices have recently become available that also measure red-edge (730 nm) reflectance. We tested the hypothesis that the additional availability of red-edge reflectance information would improve AGORS of plant stress induced chlorophyll breakdown in Scots pine (*Pinus sylvestris*). Our results showed that the availability of red-edge reflectance information improved AGORS estimates of stress induced variation in chlorophyll concentration (r^2^ > 0.73, RMSE < 1.69) when compared to those without (r^2^ = 0.57, RMSE = 2.11).

## 1. Introduction

Plant stress adversely affects container seedling quality, productivity, and thus nursery profit margins. Early detection of seedling stress is of interest to nursery managers because it facilitates corrective changes to cultural practices before stress is well established and thus reduces the risk of financial loss [1]. Many nurseries visually monitor seedlings for stress symptoms during daily operations and periodic inventories, and some submit randomly selected tissue samples for nutrient analysis [2]. A possible alternative to visual plant health monitoring and tissue tests is the use of optical sensors that rely on light to assess the physiological status of plants either at the leaf or canopy level. Canopy, when compared to leaf level optical sensing, is more affected by confounding factors such as variation in biomass or soil background. However, optical sensors that operate at the canopy level have a considerably higher sampling efficiency.

Passive optical sensors traditionally used for optical plant analysis require reference measurements to standardize spectral readings to ambient light conditions. Since light conditions tend to change frequently, passive optical sensors constantly require time-consuming reference measurements, limiting their operational use. In addition, they are costly and may require special training [3]. A more cost-effective and user friendly alternative might be the use of rugged active ground optical remote sensing (AGORS) devices that are used in agriculture for variable-rate nitrogen management [4].

**Figure 1.**
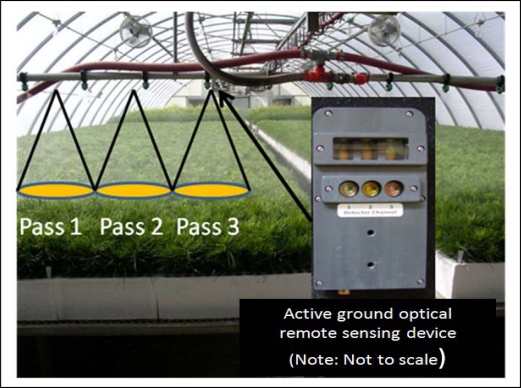
Potential operational setup of an active ground optical remote sensing device on irrigation boom (Note: active ground optical remote sensing device not to scale).

Light emitting diodes in the AGORS devices actively emit modulated light and integrated photodetectors measure only reflected light that has the sensor specific modulation frequency. Sunlight is not measured by the photodetectors since it is not modulated. These technical properties of the AGORS devices allow them to take up to 100 spectral measurements per second, independent of ambient light conditions and without the need for time consuming spectral reference readings. Hence, AGORS devices could autonomously measure canopy reflectance from irrigation booms on-the-go, independent of light conditions (Figure 1). To objectively evaluate the physiological status of plants, AGORS relies on spectral indices that combine canopy reflectance values measured at different wavelengths. Wavelengths in the visible domain (400–700 nm) have proven to be most suitable to spectrally detect plant stress that is due to disease, nutrition, pests, and anthropogenic pollution [5,6]. This has been explained by the fact that most plant stress leads to the breakdown of chlorophyll, which dominates the properties of foliar reflectance throughout the visible domain [5,7–9].

A wide variety of spectral indices have been successfully used to spectrally detect variations of chlorophyll at the leaf level [10]. However, isolating the spectral chlorophyll signal at the canopy level has proven to be more challenging due to a complex interaction of spectral signals that compose the canopy spectral response [4,11]. Traditionally, AGORS relied solely on the red (590–670 nm) and near-infrared (>760 nm) wavebands to measure the Normalized Difference Vegetation Index (NDVI) [12].The latter index is reported to be highly sensitive to variations in biomass or Leaf Area Index (LAI), yet insensitive to chlorophyll a+b concentration (Chl_ab_) [13]. Nevertheless, NDVI has been shown to be correlated with Chl_ab_ because Chl_ab_ often co-varies with biomass [14].

Recently, an AGORS device (ACS-470, Holland Scientific, Inc., Lincoln, NE, USA) has become commercially available that provides additional red-edge (700–740 nm) reflectance information. Recent research with passive optical sensors suggests that red-edge reflectance information provides important spectral information to remotely predict Chl_ab_ [11,13,15]. However, relatively little is known about whether the additional availability of red-edge waveband reflectance information improves AGORS of seedling stress. Our objective in this study was to test the hypothesis that the additional availability of red-edge waveband reflectance information would improve AGORS of seedling stress by means of nursery-grown Scots pine (*Pinus sylvestris*) seedlings.

## 2. Methods

### 2.1. Study Design and Logistics

A total of 945 Scots pine seedlings were sown on 12 February 2008 into 21 Copperblock™ 615A containers (Beaver Plastics, Acheson, Alberta, Canada) at the University of Idaho Center for Forest Nursery and Seedling Research in Moscow, Idaho. Copperblock™ 615A containers have 45 cavities per container, with a cavity volume of 340 mL and a density of 213 cavities m^−2^. Seedlings were grown in a greenhouse for two months and were then transferred outside where they received ambient precipitation plus additional irrigation as necessary to maintain 80–85% water content, expressed as a percentage of total container weight. The growing substrate used was a mixture of equal parts of vermiculite and forestry grade peat moss (Sun Gro Horticulture Distribution Inc., Bellevue, WA, USA). Chl_ab_ of tree seedlings was manipulated to simulate stress induced chlorophyll breakdown by applying one of seven different fertilizer rates of controlled-release fertilizer (2.5, 5, 7.5, 10, 12.5, 15, and 17.5 kg m^−3^) to each container with three replications per fertilization treatment. The fertilizer used was Osmocote® Exact Lo-Start 18N-6P-12K (Scotts-Sierra Horticultural Products Company, Marysville, OH, USA), which has a 14–16 month release time at a growing medium temperature of 21 °C.

### 2.2. Measurements

Measurements were conducted before full canopy closure. The moisture level of the soil was constant between samples. Spectral reflectance measurements were obtained on 11 September 2008 with the ACS-470 (Holland Scientific, Inc., Lincoln, NE, USA). The AGORS device was mounted 0.85 m above the container which resulted in a beam width of 0.48 m and length of 0.08 m, which allowed approximately 7 trees to be measured at a time. The spectral measurements for each container were each calculated as the mean of 50 spectra taken from the same viewing position. The ACS-470 bands corresponded with the following wavelengths: band 1 (659–681 nm), band 2 (720–740 nm), and band 3 (>760nm). From the spectral measurements, the red-edge reflectance employing Normalized Difference Red Edge Index (NDRE) [15] and NDVI were calculated as well as their ratio (NDRE/NDVI) [15] which is known as the Canopy Chlorophyll Content Index (CCCI) (Table 1). Combined indices, such as the CCCI, have been proposed to increase sensitivity of remote predictions of Chl_ab_ as well as resistance to variation in biomass or LAI [4,11]. In principle, the spectral index in the denominator accounts for the variability of the spectral index in the numerator that is not caused by variations in chlorophyll concentration.

To quantify seedling characteristics, the tree seedling located in the center of the ACS-470 beam was cut at the base and the height (nearest 1 mm) and the fresh biomass weight (nearest 0.01 g) of the seedling was measured. The chlorophyll concentration of needles was determined by randomly sampling 0.1 g of needle mass and cutting the sampled needles into fine pieces (< 0.5 × 0.5 mm). The cut needles were placed into 10 mL of aqueous 80% acetone, ground, and stored in a dark room for 24 hours. Chlorophyll extracts were then placed in a centrifuge for 5 minutes and the absorbance of a 3 mL subsample was measured at 644 nm and 663 nm with a Thermo Scientific GENESYS 20™ visible spectrophotometer (Thermo Fisher Scientific Inc., MA, USA). The Chl_ab_ of the chlorophyll extract solution was calculated according to Lichtenthaler [16]. All Chl_ab_ values were calculated as mg of total chlorophyll a+b per g fresh weight (mg g^−1^).

### 2.3. Statistical Analysis

Simple linear regression was performed in the open-source software package, R 2.8.1 (R Development Core Team, 2008) to examine the relationship between each spectral index and measured Chl_ab_ (n = 21). Goodness of fit was evaluated based on the coefficient of simple determination (r^2^). The quality of the regression models was assessed using leave-one-out cross-validation, in which each data point is iteratively omitted, the model is re-fitted, and the resulting regression used to predict the omitted data point. Based on the resultant Chl_ab_ predictions, the root mean square error (RMSE), the r^2^, and the slope and intercept could be calculated by relating observed (dependent variable) to predicted (independent variable) Chl_ab_. A RMSE value of 0.0 and an r^2^ value of 1.0 indicates high precision and a slope of 1.0 and intercept of 0.0 high accuracy [17].

## 3. Results and Discussion

**Figure 2.**
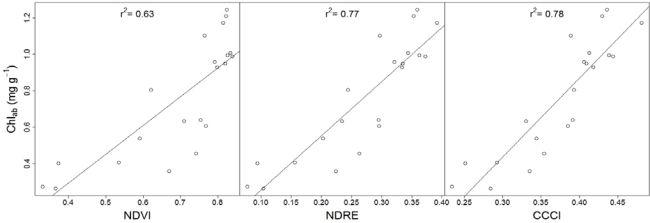
Relationship between Chl_ab_ and spectral indices.

Slight differences in greenness amongst seedlings of different fertilizer treatments were visible. Across all treatments, the average Chl_ab_ was 0.76 ± 0.32 mg g^−1^ (mean ± standard deviation), the average seedling height was 93.3 ± 46.2 mm, and the average fresh biomass weight was 8.63 ± 4.75 g. The red-edge reflectance employing CCCI and NDRE showed a stronger linear correlation (r^2^ = 0.78 and 0.77, respectively) with Chl_ab_ than non red-edge reflectance employing NDVI (r^2^ = 0.63; Figure 2). It is important to note that though a non-linear regression model between Chl_ab_ and NDVI would have resulted in a slightly stronger relationship, the higher r^2^ would have suppressed the low sensitivity of NDVI to high Chl_ab_ (>0.6 mg g^−1^).

Some of the scatter across the regression lines might be explained by variability in background reflectance [4]. In cases where variability in background reflectance is high it might become more advantageous to use combined instead of single indices; Eitel *et al.* [5] showed for example that combined indices were less affected by variations in soil reflectance than single indices.

Since early stress detection requires spectral indices to be sensitive to variations in high Chl_ab_ [10], we reanalyzed the data after removing low Chl_ab_ values (<0.6 mg g^−1^). Not surprisingly, the strength of the spectral index to Chl_ab_ relationships decreased upon removal of low Chl_ab_ values. However, it is important to note that the strength of the spectral index to Chl_ab_ relationship decreased less for red-edge reflectance employing CCCI (r^2^ = 0.53) and NDRE (r^2^ = 0.54) when compared to NDVI (r^2^ = 0.34). This finding suggests that the red-edge reflectance employing CCCI and NDRE are more suitable to detect early stages of stress induced chlorophyll breakdown than the non red-edge reflectance employing NDVI.

**Figure 3.**
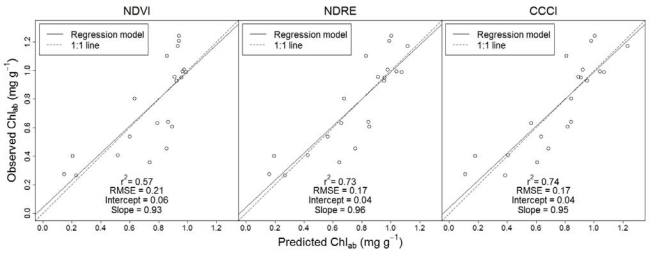
Cross validation results showing relationships between predicted and observed Chl_ab_ concentration for regression models using NDVI, NDRE, and CCCI, respectively, as predictor variables.

The results of the cross-validation further suggested the superior performance of the red-edge reflectance employing indices: regression models employing CCCI and NDRE provided more precise Chl_ab_ predictions (r^2^ ≥ 0.73 and RMSE = 0.17) than did the regression model employing NDVI (r^2^ = 0.57 and RMSE = 0.21) (Figure 3). Regression models employing CCCI and NDRE also provided more accurate Chl_ab_ predictions (intercept = 0.04/slope = 0.95 and intercept = 0.04/ slope = 0.96) than the regression model employing NDVI (intercept = 0.06/slope 0.93).

## 4. Conclusions

The results presented here suggest that availability of red-edge waveband reflectance information improves AGORS of plant stress, particularly at early stages, and thus support our hypothesis. To operationally use AGORS devices in a nursery setting, we envision that AGORS devices linked with either a combined rotary encoder and measuring wheel [18], or optoelectronic non-contact linear encoder positioning device, could be mounted on automated overhead irrigation boom systems to acquire spatially referenced spectral indices on-the-go (Figure 1). The spatially referenced spectral indices could then be used to locate seedlings with low Chl_ab_ that need attention. Furthermore, spatial patterns in the Chl_ab_ of seedlings, due to non-uniform irrigation spray nozzle performance, local variation in temperature or air circulation within the greenhouse, and other growth factors, could also be identified and mapped. However, it is important to note that although spectral indices can help to detect and monitor seedling quality, they do not provide information about the specific causes of plant stress.

Since the width of the sensor's field of view will likely not cover the entire width of a growing table with one boom pass, several passes might be necessary, with the sensor’s location along the boom advanced after each pass. Further studies with other plant species are necessary to verify our results and evaluate the operational use of AGORS in nursery settings.

## Figures and Tables

**Table 1.** Spectral indices used in this study. Band #’s are sensitive to the following wavelengths: band 1 (659–681 nm), band 2 (720–740 nm), and band 3 (>760 nm).

**Vegetation Index**	**Equation**	**Reference**
	*Structural Index*	
Normalized Difference Vegetation Index (NDVI)	NDVI = (R_band3_ − R_band1_)/(R_band3_ + R_band1_)	[12]
	*Chlorophyll Index*	
Normalized Difference Red Edge Index (NDRE)	NDRE = (R_band3_ − R_band2_)/(R_band3_ + R_band2_)	[15]
	*Combined Index*	
Canopy Chlorophyll Content Index (CCCI)	CCCI = NDRE/NDVI	[15]
